# Continuous Education of Basic Life Support (BLS) through Social Media; a Quasi-Experimental Study

**Published:** 2019-01-20

**Authors:** Seyyed Mahdi Zia Ziabari, Vahid Monsef Kasmaei, Lida Khoshgozaran, Maryam Shakiba

**Affiliations:** 1 Department of Emergency Medicine, School of Medicine, Guilan University of Medical Sciences, Rasht, Iran.; 2 Guilan Road Trauma Research Center, School of Health, Guilan University of Medical Sciences, Rasht, Iran

**Keywords:** Education, cardiopulmonary resuscitation, mobile application, computer-assisted instruction

## Abstract

**Introduction:**

It is imperative, if not fundamental, for medical students to receive continuing education in Basic life support (BLS). This present study aimed to investigate the effectiveness of continuing education using social media in this regard.

**Methods:**

In this quasi-experimental study, a questionnaire containing 20 items about BLS awareness was completed by the interns who were trained monthly in the emergency medicine training course. Then half of the interns received distant education through Telegram software up to three months, and the other did not receive any further education. Again, both groups completed the questionnaire at the end of the third month.

**Results:**

100 medical interns with the mean age of 25.05 ± 1.26 years were allocated to two groups of 50 subjects (65.0% female). Two groups were similar regarding the mean age (p = 0.304), gender (p = 0.529), mean awareness score at the beginning of the study (p = 0.916), and average pre-internship exam score (p = 0.080). The mean awareness score of medical interns increased significantly in intervention (11.44 ± 2.37 to 14.88 ± 1.97, p < 0.0001) and control (11.38 ± 3.22 to 12.54 ± 3.04, p < 0.0001) groups at second examination. But mean difference of awareness score, before and after the education, was significantly higher in intervention group (3.44 ± 1.48 versus 1.16 ± 1.51; p < 0.0001).

**Conclusion:**

The increase in the BLS awareness score was significantly higher in medical interns who were continuously educated using Telegram social media software.

## Introduction

Despite many advances in medicine, sudden cardiac arrest remains a leading cause of death in most countries worldwide (1). Subsequent outcomes of cardiac arrest depend on basic measures such as chest compression, defibrillator, and more advanced actions (2). The importance of cardiopulmonary resuscitation (CPR) has been well documented, so that a good CPR can reduce mortality up to 50% (3).

In general, the ability to diagnose and treat a respiratory or cardiac arrest is a basic medical skill that all health care providers including medical students should have these skills. They should learn to carry out CPR and pre-hospital activities and increase their skills during educational courses (4, 5).

The measures for basic life support (BLS) for adolescents outside the hospital include rapid detection of cardiac arrest, quick activation of the emergency medical services (EMS), pulse check, CPR, and rapid defibrillation with an automated external defibrillator (AED) (6).

Since the quality of CPR depends on the learner memorizing and applying the scientific and practical skills, continuing education should be provided in this regard (7).

The use of e-learning in medical education has had good results and has been effective (8, 9). In this way, electronic tools and software are used as a means to improve learning, communication and interactions, and increasingly to train medical professionals (10, 11). The advancement in e-learning technology and the use of computers and online training has made it possible to save costs and transfer data without any time limit. Also, the person-to-person training capabilities meet the learner's needs well (12, 13).

Although the BLS course is held every two years, researches have shown that quality of BLS clinical skills begin to decrease after 3 months (14-16).

Studies showed that the students had more tendency and self-confidence to perform CPR after multiple educational sessions (17, 18). Researches have also shown that watching an educational video clip on cell phone to remind and review BLS content increases the skill and self-confidence to carry out CPR and the use of AED in rescuers (18).

Considering the above mentioned, we decide to investigate the effectiveness of continuous education of BLS through social media (Telegram software) on medical interns.

## Methods

***Study design and setting***

This quasi-experimental study, was conducted on medical interns who were trained monthly in the emergency medicine training course of Poursina Hospital, Rasht, Iran, from August 2016 to July 2017. All medical interns entered the study with informed consent, and reviewing the research project was carried out by the Ethics Committee of the Research Unit of Guilan University of Medical Sciences (IR. GUMS.REC.1396.69). the study was registered in Iranian Registry of clinical trials with number: IRCT20110818007369N5.

***Participants***

All medical interns who were passed the one month emergency medicine educational program in mentioned hospital during the study period were enrolled. Dissatisfaction with continuing to participate in the study, lack of access to smartphone, not using of Telegram software were among the exclusion criteria. There was no any sex or age limitation. The medical interns were given an ethical commitment that their personal information would remain confidential and they also gave a moral commitment to not share the contents of the classroom and the software until the end of the study with other interns.

***Data gathering***

To assess the students' awareness regarding the BLS at the beginning and end of the study, a questionnaire containing 20 items was used (19). The Persian version of this questionnaire was translated into English, and it was again translated into Persian by a native. To determine the quantitative validity, content validity ratio (CVR) and content validity index (CVI) were used. The score for cut-off point in Lawshe table for 10 experts was 0.62. In the questionnaire, the CVR scores of all questions were 0.8 to 1, which is confirmed in terms of CVR index. Also, in terms of the CVI, the lowest CVI was 0.7 and its maximum was 1 in the three dimensions of relevance, simplicity and clarity. Therefore, questions with a CVI of between 0.7 and 0.8 were subject to partial review, and the remaining questions due to the high CVI remained unchanged. To determine the internal consistency of the questions, the Richardson coefficient 20 was used, with the obtained alpha value equal to 0.996 (more than 0.7), indicating the internal consistency of the questions in the calculation of students' awareness to the BLS algorithm.

***Intervention***

Mentioned questionnaire was first distributed among medical interns, and they were asked to answer the questions based on their information and to enter data including their name, age, gender, and average pre-internship exam score in the questionnaire. 

Based on the answers to the questions, a score of 1-20 was attributed to the students (correct answer to each question had 1 score, the wrong answer was zero, and negative score did not belong to the wrong answers). The scores obtained from the questionnaire were gained with algebraic scores from each question (zero or one), and the students' awareness was measured based on these scores. 

After completing the questionnaire, all of the interns received an in-person tutorial using PowerPoint software by the teachers of Rasht Poursina hospital, and an emergency medicine resident, based on the 2015 AHA educational guideline on BLS for adults and children (1, 6, 20-22).

After being trained at the hospital, half of the interns who were selected sequentially and non-randomly, received distant continuing education for up to three months through social media (Telegram software, which is comprehensive and accessible in Iran). This training was conducted in a person-to-person manner by sending abstracts of taught materials in the classroom based on the 2015 AHA guidelines, which was presented in short texts, photographs and educational videos related to how to perform BLS.

The other half of the participants did not receive any further training after the in-person education offered at the hospital.

Then, at the end of the third month after holding the first in-person class, both groups received the questionnaire through the Telegram software, and the awareness on BLS was measured by refilling the same questionnaire.

***Statistical analysis***

We calculated sample size using altman monogram with α: 0.01, β: 0.2 and d²: 0.9. The allocation ratio was 1:1, and finally 50 students entered in each group. 

Data were analysed using mean and standard deviation. Normality of data was evaluated using skewness and kurtosis indices. Paired t-test and independent t-test were used to compare means between the groups. All analyses were performed in SPSS version 19 and a significant level was considered as below 0.05.

## Results

119 medical interns were studied. 19 out of 119 students were excluded (3 were not using the Telegram software, 4 were not satisfied to participate to the study, 12 did not fill the questionnaire after 3 months). Finally 100 medical interns with the mean age of 25.05 ± 1.26 (23 - 31) years were allocated to two groups of 50 subjects (65.0% female). 

Table 1 compare the baseline characteristics of two groups. Two groups were similar regarding the mean age (p = 0.304), gender (p = 0.529), mean awareness score at the beginning of the study (p = 0.916), and average pre-internship exam score (p = 0.080). 

**Figure 1:**
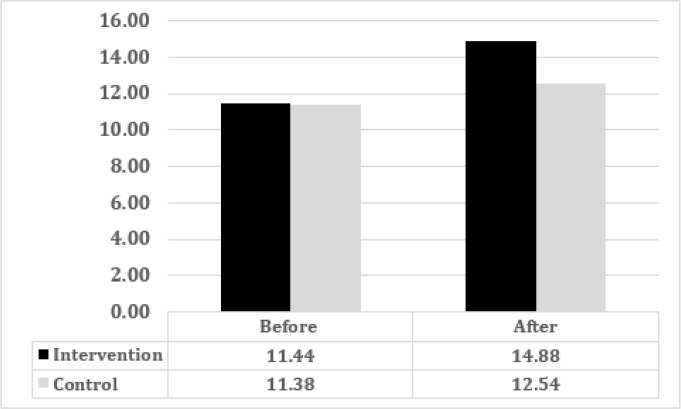
Mean awareness score in intervention and control groups before and after the education (p < 0.0001)

The mean awareness score of medical interns increased significantly in intervention (11.44 ± 2.37 to 14.88 ± 1.97, p < 0.0001) and control (11.38 ± 3.22 to 12.54 ± 3.04, p < 0.0001) groups at second examination (figure 1). But mean difference of awareness score, before and after the education, was significantly higher in intervention group (3.44 ± 1.48 versus 1.16 ± 1.51; p < 0.0001).

## Discussion

The findings of this study showed that the increase in the BLS awareness score was significantly higher in medical interns who were continuously educated using Telegram social media software. 

**Table 1:** Basel line characteristics of case (continuous education with social media) and control (regular education) groups

**Variable**	**Value**	**P values**
**Gender (male/female ratio)**		
Case	31/19 (1.63)	0.529
Control	34/16(2.12)	
**Age (year)**		
Case	24.92 ± 1.01	0.304
Control	25.18 ± 1.46	
**Awareness score**		
Case	11.44 ± 2.37	0.916
Control	11.38 ± 3.22	
**Pre-internship exam score**		
Case	15.97 ± 0.81	0.080
Control	16.24± 0.71	

Data are presented as mean ± standard deviation.

An interventional study by Khorasani et al. showed that the mean score of awareness and practice before and after the primary emergency care education workshops indicated a significant difference in practice before and after taking part in workshop in dentistry interns (23). 

Ji Yun Ahn et al. showed that watching an educational video clip in cell phone in order to remind and review the BLS materials increased skill and self-confidence to perform CPR and using AED in rescuers after three months (18). In our study, the impact of this continuous education was seen positively, although there was no need to use a memory card and a reminder message to view clips and educational pictures, and all interns could have enough time to read, understand, and answer person-to-person questions using Telegram software within three months.

In a randomized controlled trial, Eric L. Einspruch et al. in the United States, compared memorizing CPR skills in conventional education to teaching through 22-minute self-learning videos. In both groups, the decrease amount in skills after two months was generally the same. In the second assessment two months later, the subjects of the self-learning group were better than the conventional group in all skills, except for the emergency call, which were the same for both groups. In the assessment with manikin, the self-learning group was better than the conventional training group in the ventilation volume, but both groups performed equally in proper hand positioning in chest compression and massage depth (7). Contrary to this study showing a decrease in skills after two months, the awareness of interns’ after three-month continuing education did not reduce, but increased significantly compared to the control group in our study.

In general, the ability to diagnose and treat a respiratory or cardiac arrest is a basic medical skill that all medical students should have the skills. They should learn CPR and pre-hospital activities and increase their skills during educational courses (4, 5). Given that these skills and awareness about how to conduct appropriate BLS are forgotten over time, and according to AHA 2015, studies have demonstrated that deterioration of BLS skills in as little as 3 months after initial training (14-16), therefore, it should be emphasized that in order to maintain and improve clinical skills, they need to be adequately memorized with repeated training (7).

With the help of information and communication technology capabilities, students are able to provide information at any time and place quickly. The learner can have enough time to read, understand, and answer the questions in a person to person manner, in spite of his/her work, family, disability and geographical location; this encourages and motivates him/her (24).

Considering the widespread use of modern Telegram software in society especially in the student population, this study examined the effect of person to person and continuing education of CPR operation using Telegram among medical students. Since all students were spending their internship in different parts, the software provided continuous communication with each other.

***Limitation***

The weaknesses of this study were the lack of randomization and the impossibility of blinding due to the nature of the study, and the lack of facilities for examining the practical skills of students at the same time. Since it is not possible to examine skills in addition to knowledge, it is suggested that future studies consider these two issues together.

## Conclusion:

The results of this study indicate the positive effect of continuing education of BLS through Telegram software on the awareness of medical students. This finding can demonstrate the positive role of using modern communication networks and the possibility of using them in the student community.
